# The Combined Inoculation of *Curvularia lunata* AR11 and Biochar Stimulates Synthetic Silicon and Potassium Phosphate Use Efficiency, and Mitigates Salt and Drought Stresses in Rice

**DOI:** 10.3389/fpls.2022.816858

**Published:** 2022-03-03

**Authors:** Arjun Adhikari, Muhammad Aaqil Khan, Muhammad Imran, Ko-Eun Lee, Sang-Mo Kang, Jin Y. Shin, Gil-Jae Joo, Murtaza Khan, Byung-Wook Yun, In-Jung Lee

**Affiliations:** ^1^Department of Applied Biosciences, Kyungpook National University, Daegu, South Korea; ^2^Department of Chemistry and Environmental Science, Medgar Evers College, The City University of New York, New York City, NY, United States; ^3^Institute of Agricultural Science and Technology, Kyungpook National University, Daegu, South Korea

**Keywords:** abiotic stress, environment, fertilizer, nutrient, physiology, PGPMs

## Abstract

Synthetic chemical fertilizers are a fundamental source of nutrition for agricultural crops; however, their limited availability, low plant uptake, and excessive application have caused severe ecological imbalances. In addition, the gravity of environmental stresses, such as salinity and water stress, has already exceeded the threshold limit. Therefore, the optimization of nutrient efficiency in terms of plant uptake is crucial for sustainable agricultural production. To address these challenges, we isolated the rhizospheric fungus *Curvularia lunata* ARJ2020 (AR11) and screened the optimum doses of biochar, silicon, and potassium phosphate (K_2_HPO_4_), and used them—individually or jointly—to treat rice plants subjected to salt (150 mM) and drought stress (20–40% soil moisture). Bioassay analysis revealed that AR11 is a highly halotolerant and drought-resistant strain with an innate ability to produce gibberellin (GA_1_, GA_3_, GA_4_, and GA_7_) and organic acids (i.e., acetic, succinic, tartaric, and malic acids). In the plant experiment, the co-application of AR11 + Biochar + Si + K_2_HPO_4_ significantly improved rice growth under both salt and drought stresses. The plant growth regulator known as abscisic acid, was significantly reduced in co-application-treated rice plants exposed to both drought and salt stress conditions. These plants showed higher Si (80%), P (69%), and K (85%) contents and a markedly low Na^+^ ion (208%) concentration. The results were further validated by the higher expression of the Si-carrying gene *OsLSi1*, the salt-tolerant gene *OsHKT2*, and the *OsGRAS23*’s drought-tolerant transcriptome. Interestingly, the beneficial effect of AR11 was significantly higher than that of the co-application of Biochar + Si + K_2_HPO_4_ under drought. Moreover, the proline content of AR11-treated plants decreased significantly, and an enhancement of plant growth-promoting characteristics was observed. These results suggest that the integrated co-application of biochar, chemical fertilizers, and microbiome could mitigate abiotic stresses, stimulate the bioavailability of essential nutrients, relieve phytotoxicity, and ultimately enhance plant growth.

## Introduction

Sustainable agricultural practices have been recognized as a pragmatic economic approach to attain the goal of food security (Francis and Porter, 2011). However, one of the biggest challenges for the sustainability transitions is the management of chemical fertilizers and environmental stresses, such as drought and salinity (Butcher et al., 2016; Mark Ibekwe et al., 2017). Salinization-related reduction in crop yield in semiarid and arid region of world accounts to 18–43% and more than three percent of soil resources are affected globally (Singh, 2021). Similarly, the percentage of lands affected by drought has doubled in the last 40 years, affecting farmers more than any other natural hazards (FAO, 2021). It is predicted that, by the first half of the twenty first century, water drought events will dramatically worsen, reaching record levels never seen before in the history of mankind (Premanandh, 2011). Forecasts predict a 1.3-fold increase in water requirements in the agricultural sector by 2025 (Premanandh, 2011). In addition, the world’s population is rapidly increasing and is expected to reach nine billion by 2050 (Fariduddin et al., 2019). In these scenarios, the mitigation of salt stress and droughts is crucial to overcome the probable risk of food insecurity (Zhai et al., 2019).

Understanding the physiological context of salt and water deficiency stresses is essential for their alleviation and to develop long-term management strategies (Dawood et al., 2021). In general, salt and drought stresses harm plant metabolism, causing ionic imbalance, osmotic stress, oxidative damage, damage of cellular structures, and a decrease in gas exchange rates, which ultimately lead to plant death (Bhusal et al., 2021; Khan et al., 2021; Nabi et al., 2021).

Plants acquire several strategies to cope with abiotic stresses and, among these, ionic adjustment such as Na^+^/K^+^ ratio, and endogenous phytohormones such as ABA regulation play a key role in modulating the overall physiological system (Garg and Bhandari, 2016; Zaid et al., 2021). Under salinity stress, plants accumulate toxic ions which causes ionic toxicity; in response, amassing of soluble osmolytes and synthesis of reducing agents occur which aids to osmotic adjustment and nutrients balance (Golldack et al., 2014; Dawood et al., 2021). Under drought stress, plants mobilize the ABA channel that activates guard cells to regulate stomatal conductance and sustain high moisture level, by influencing the leaf and soil water potential (Harb et al., 2010). It has been reported that the plants’ ionic system is extensively regulated by the balanced presence of sodium, potassium, phosphorus, and silicon (Etesami, 2018). Bargaz et al. (2016) showed that the phosphorus (P) supply induced salt tolerance in *Phaseolus vulgaris* through the acquisition of K^+^ and Ca^++^. Similarly, the role of silicon (Si) in improving plant physiology, growth, and yield has been widely reported in numerous studies (Kim et al., 2014; Zhu and Gong, 2014). The enhancement of P, K, and Si uptake in plants was shown to mitigate several stresses in plants, including both biotic (e.g., pathogens) (Bakhat et al., 2018), and abiotic ones, such as heavy metal stress, water stress, thermal stress, and salt stress (Zaid et al., 2018). Therefore, it can be concluded that the proper utilization of Si, P, and K could improve plant metabolism and growth under diverse environmental conditions.

The nutritional sources of plants largely depend on synthetic fertilizers. According to the Food and Agriculture Organization (FAO) (Food and Agriculture Organization of the United Nations, 2017), the world consumption of nitrogen, phosphorus, and potassium (NPK) was estimated at about 186.67 million tonnes in 2016, and P demand specifically increased by 2.2% in 2020, but the utilization efficiency by plants is limited to 10–15% (Simpson et al., 2011). Soil nutrient depletion has been adversely affecting poverty-stricken farmers worldwide (Yang et al., 2009). The net accumulation of toxic elements in agriculture is increasing due to the overuse of synthetic chemicals and to industrialization, which result in water eutrophication and environmental hazards (Simpson et al., 2011; Bouraoui and Grizzetti, 2014; Ali et al., 2019). The scientific community has repeatedly warned that the long-term, continuous application of chemical fertilizers without adequate organic amendments may degrade soils to such an extent that they may become extremely low or non-responsive to inorganic fertilizers (Fofana et al., 2008; Tittonell and Giller, 2013; Tounkara et al., 2020).

Plant growth-promoting microorganisms (PGPMs) and biochar are the emerging alternatives to agrochemicals for the production sustainable food (Yang et al., 2009; Abhilash et al., 2016; Ali et al., 2017). PGPMs have been widely reported to enhance plant growth-promoting traits such as through abscisic acid (ABA) regulation (Cohen et al., 2009), gibberellin (GA) production (Bottini et al., 2004), and phosphate, potassium, and silicate solubilization (Adhikari et al., 2020a). Similarly, biochar has been widely reported to facilitate PGPM growth, prevent disease incidence, stabilize heavy metal concentrations, increase soil fertility, and mitigate severe biotic and abiotic stresses (Farhangi-Abriz and Torabian, 2017; Gavili et al., 2019). Under stress conditions, these biostimulants enhance plant physiology through the modulation of phytohormones, including ABA, jasmonic acid (JA), gibberellic acid (GA), and salicylic acid (SA) (Waqas et al., 2012). In addition, they were reported to strengthen the antioxidant system through the modulation of amino acids under stress (Qadir et al., 2020). Moreover, biochar and PGPMs were shown to have a functional role in lowering Na^+^ uptake and enhancing K^+^ ion uptake in plants exposed to salt stress, while also enhancing the water holding capacity of soils and plants under drought stress (Ali et al., 2017; Khan et al., 2019a).

Salinity and drought were shown to be crucial limiting factors for the growth of major cereal crops such as rice (Kumar et al., 2013; Tang et al., 2019). Rice is the most important staple crop, consumed by billions of people globally, representing 75% of the total calorie intake in Asian countries (Xia et al., 2019). As mineral elements—such as Si, P, and K—play a key role in the increase of several biotic and abiotic stresses, an efficient strategy to boost their uptake efficiency in plants is still lacking. Hence, there is an urgent call for developing a sustainable agriculture contrivance in perspective of climate change, abiotic stresses, excess fertilizer demand, environmental pollution and high-production cost. To address these challenges, in the present study, we identified a highly drought- and salt-tolerant fungus and assessed its plant growth-promoting traits. The effects of this fungal species on the modulation of rice physiology under salt and drought stresses were elucidated along with those of biochar, inorganic silicon, phosphorous, and potassium.

## Materials and Methods

### Fungus Isolation and Identification

**FIGURE 1:**
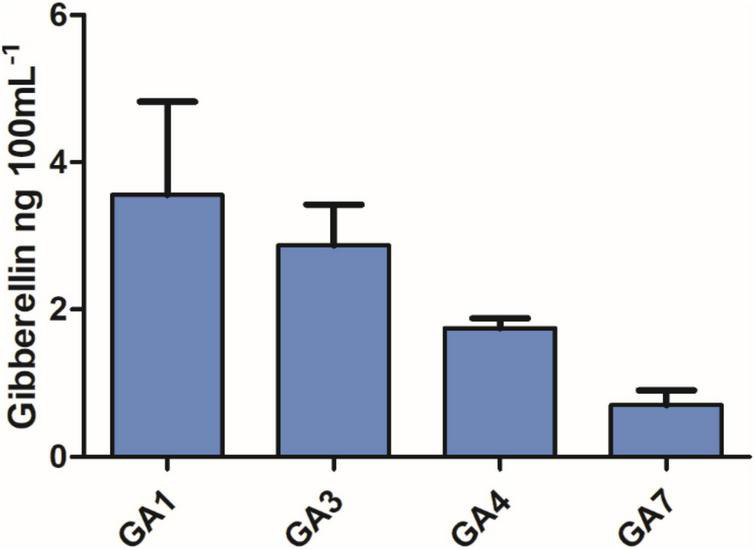
Quantification of gibberellin content in the *Curvularia lunata* (AR11) pure culture.

A total of 120 different fungal colonies were isolated from the agriculture waste decomposing site at Kyungpook National University, Daegu, and their pathogenicity and plant growth-promoting traits were evaluated (Supplementary Table 1 and Figure 1). The GA production of the isolates was tested through inoculation on the GA-deficient dwarf mutant rice strain *Waito-C*, in glucose agar media. The AR11 strain, which was shown to highly influence growth performance in *Waito-C* plants, was selected for further experiments (Supplementary Figure 2). The identification of AR11 was performed by sequencing the internal transcribed regions (ITS) using universal primer with ITS1 (5′-TCCGTAGGTGAACCTGCGG-3′) and ITS4 (5′-GCTGCGTTCTTCATCGATGC-3′).

### Assessment of the Plant Growth Promoting-Traits of AR11

#### Growth Performance Test Under Stress

The AR11 fungal strain was grown under salt concentrations of 100, 200, 300, 400, and 500 mM; and at a polyethylene glycol (PEG 6000)-supplemented potato-dextrose Agar (PDA) media. The isolate growth was observed with different osmotic potentials (−0.05, −00.15, −00.3, −0.49, and −00.73 MPa). In addition, fungal growth on inorganic Si and K_2_HPO_4_ was also evaluated.

#### Gibberellic Acid, Organic Acids, and Amino Acid Quantification

• GA: GA was quantified based on the method described by Lee et al. (2015). In brief, 200 mL of 1-week-old AR11 culture broth was supplemented with the GA internal standard (^2^H_2_), and extraction was performed. The extract obtained was then subjected to chromatography and mass spectroscopy for GA detection and quantification.
• Organic acids: Organic acids, such as succinic, malic, acetic, and tartaric acid, were quantified based on the method described by Lee et al. (2019). In brief, after the fungal suspension culture was grown in a shaking incubator for a week, it was centrifuged and the supernatant was collected, filtered, and injected on high-performance liquid chromatography (HPLC, Waters 600, Milford, MA, United States) using a PL Hi-Plex H column (7.7 × 300 mm, Waters Co., Milford, MA, United States), detector refractive index (RI, Waters 410, Milford, MA, United States), and 5 mM H_2_SO_4_ was used as solvent in distilled water.
• Amino acids: Free amino acids were quantified on a 1-week-old fungal pure culture; the culture was centrifuged, and the supernatant was separated. The solution was then filtered and suspended with 5% trichloroacetic acid and was subjected to shaking for 2 h. The solvent was further centrifuged, and the supernatant was separated, filtered, and injected on an amino acid analyzer (L-8900; Hitachi, Tokyo, Japan).

### Plant Material and Experiment

Two independent experiments were conducted on salt and drought stresses in rice. The experimental design is represented in Table 1. The statement of results is mostly written with the data compared with T2 (Control +ve) in the results section.

**TABLE 1:** Outline of the experimental design for the independent experiment conducted under salt and drought stress conditions.

Treatment	Salt stress	Drought stress
T1	Control –ve (-S)	Control1 –ve (-D)
T2	Control +ve (-S)	Control2 +ve (-D)
T3	Si (-S)	Si (-D)
T4	KP (-S)	KP (-D)
T5	AR11 (-S)	AR11 (-D)
T6	Bio (-S)	Bio (-D)
T7	Si + KP (-S)	Si + KP (-D)
T8	Si + KP + AR11 (-S)	Si + KP + AR11 (-D)
T9	Si + KP + Bio (-S)	Si + KP + Bio (-D)
T10	Si + KP + AR11 (-S)	Si + KP + Bio + AR11 (-D)

*(-S), salt stressed; (-D), drought stressed; −ve, only water (no stress); +ve, complete stress (no treatment); Si, silicon; KP, potassium phosphate (K_2_HPO_4_); AR11, Curvularia lunata AR11 fungal suspension; Bio, biochar.*

#### Soil and Pot Preparation

Before transplanting the plants, the soil was prepared, based on the above-mentioned experimental design. The screening of plant growth was performed with randomly selected, similar concentration of Si and K_2_HPO_4_ (1, 2, 3, 4, and 5 mM), and biochar (composition: charcoal 70%, Dolomite 10%, Zeolite 5%, Molasses 10%, and Peat 5%) at 1, 2, 3, 4, and 5% w/w. It was conducted under salt and drought stress conditions. The optimum dose of 2 mM for Si, 3 mM for K_2_HPO_4_ and 3% for biochar was selected based on plant growth observations. The biochar and fungal suspension were added before the transplantation of seedlings, whereas Si and K_2_HPO_4_ were supplied after it. Paddy soil (Peat moss: 5%, Cocopeat: 35.6%, Zeolite: 5%, Diatomaceous soil: 3%, Red clay: 8%, Fly ashes: 3%, Vermiculite: 40%, Fertilizer: 0.32%, pH: 0.05%, wetting agent: 0.03%, Manufacturer: Non-ghyup, Gyeongi-do, South Korea) was autoclaved twice and placed in a sealed bucket. Then, it was mixed with biochar and a quantity of 300 g soil was then transferred into each pot (diameter 3.93” × height 3.74”). The AR11 suspension was inoculated on the soil based on the previously mentioned Table 1. The soil was irrigated with autoclaved distilled water and kept in a growth chamber for 2 weeks.

#### Plant Experiment

The Japonica rice seeds *var.* “Jinbek” available in the Crop Physiology Laboratory of Kyungpook National University, were sterilized by washing with autoclaved distilled water containing 0.01% Tebuconazole and allowed to germinate in an incubator at 30^°^C for 4-days. The seeds were now sown in a 100 cells seedling trays using autoclaved paddy soil as mentioned in Section Soil and Pot Preparation and kept in a growth chamber for 3 weeks. Four equally sized, 3-week-old rice plants were transplanted in each pot as prepared in Section Soil and Pot Preparation. In the plant experiment, each treatment had eight replicates. After a week, 100 mL of Si (2 mM) and K_2_HPO_4_ (3 mM) were applied to each pot for 2 days, and the plants were normally irrigated for a week. Subsequently, they were subjected to drought and salt stresses. A volume of 20 mL of water per pot was supplied for the first 3 days, 25 mL/pot for the second 3 days, and 30 mL/pot for the last 4 days, to specifically produce drought stress. The soil moisture level was detected 20–40%, depending upon the treatments used which was estimated using the soil pH, and humidity tester (Model DM-5, Takemura Electric Works, LTD., Tokyo, Japan). Whereas, 150 mM NaCl (50 mL/pot) was applied to each pot for 3 days to induce salt stress. Adequate water was supplied for the following 7 days, after which sampling was conducted for further biochemical analysis. The entire experiment was carried out at controlled growth chamber conditions (day/night cycle: 12 h; 28°C/12 h; 22°C; relative humidity (RH) 68%; light intensity 1,000 μmm^–2^s Natrium lamps).

### Biochemical Analysis of Plants

#### Quantification of Mineral Elements

Mineral elements (Si, P, K, Na) were quantified based on the method described by Adhikari et al. (2020a). In brief, 0.1 g of free-dried sample was suspended with HNO_3_ reagent and processed using a microwave digestion system (MDS): Ultrawave (milestone). The sample was diluted with 2% HNO_3_, and the solution obtained was injected and quantified through inductively coupled plasma mass spectrometry (ICP-MS, Optima 7300DV).

#### RNA Isolation and Quantitative Real Time-PCR Analysis

RNA isolation and quantitative real time-PCR (qRT-PCR) was performed following the method described by Al Azzawi et al. (2020). In brief, RNA was isolated using TRI-reagent; cDNA was synthesized with the RNA extract and subjected to qRT-PCR to analyze the expression of the gene *OsLSi1*, *OsHKT2*, and the *OsGRAS23*. Specifically, 1 μg RNA was used for cDNA synthesis using BioFACT RT-Kit (BIOFACT, Daejeon, South Korea) following the manufacturer’s protocol. A two-step qRT-PCR was performed with Illumina ECO system (Illumina, San Diego, CA, United States). The primers used are listed in the (Supplementary Table 2).

#### Analysis of Abscisic Acid

The method described by Shahzad et al. (2017b) was followed to extract the ABA and quantify its content. In brief, 0.5 g of lyophilized ground samples was extracted with isopropanol and acetic acid (95:5 v/v). The ABA extracts were analyzed by GC-MS/SIM (6890N Network GC System and 5973 Network Mass Selective Detector: Agilent Technologies, Santa Clara, CA, United States). The peak obtained was compared to the standard curve derived from the ABA-internal standard [(± ) −03, 5, 5, 7, 7, 7-d6].

#### Polyphenol and Proline Quantification

The quantification of proline was carried out following the method reported by Shahzad et al. (2017a) using automatic amino acid analyzer (HITACHI Corporation L-8900, Tokyo, Japan) attached to a HITACHI HPLC system (packed column with ion-exchanging resin, No. 2622 PF; 4.6 × 60 mm) and ultraviolet detector (VIS1: 570 nm, VIS2: 44 nm). Polyphenol contents were quantified based on the method described by Adhikari et al. (2018). Samples were extracted with 100% methanol and the wavelength was measured at 750nm using a spectrophotometer (Multiskan GO; Thermo Fischer Scientific, Vantaa, Finland).

### Statistical Analysis

The results were analyzed in R (version 4.0.3). A least significant difference (LSD) test (*p* < 0.05) was used to determine significant differences among treatments. The mean and standard deviation were estimated in Microsoft Excel 2019, and the data were graphically presented using GraphPad Prism (version 6.01; San Diego, CA, United States).

## Results

### Identification of Isolate AR11

Isolate AR11 was identified as a *Curvularia lunata* strain and was submitted to the NCBI database as *Curvularia lunata* ARJ2020, with accession number MZ145250.

### Evaluation of the Plant Growth-Promoting Traits of AR11

The AR11 strain was shown to be highly halotolerant, drought and heavy metal resistant, and could efficiently grow on Si and K_2_HPO_4_ supplemented media. Inoculations with this strain significantly improved the growth of the GA-deficient rice mutant *Waito-C*. Moreover, GA quantification results showed that isolate AR11 could produce biologically active gibberellin, i.e., GA1, GA3, GA4, and GA7 (Figure 1). The AR11 pure culture presented a lower amount of free amino acids, whereas organic acids—such as succinic, acetic, malic, and tartaric acids—were detected in larger quantities (Tables 2, 3). Under drought stress conditions, the AR11 strain forms a layer in the upper part of the soil which produces a mulching effect on plants, preserving the water potential and allowing the gradual release of water from the upper layer. A maximum of 40% soil moisture level was detected in AR11 treated soil, whereas on other treatment it was below 30%. The AR11 strain holds water for a long period of time and delivers it to the plant allowing it to thrive.

**TABLE 2:** Quantification of organic acid content in AR11 suspension culture.

Organic acid content (ppm)	Succinic acid	Acetic acid	Tartaric acid	Malic acid
Control	500	ND	20	ND
Control	450	ND	15	ND
Control	360	ND	18	ND
AF1	1300	600	50	40
AF1	998	435	65	25
AF1	1036	202	35	21

**TABLE 3:** Free amino acid intake potential of *Curvularia lunata* AR11.

Nomenclature	Control (μg/ml)	AR11 (μg/ml)
Phosphoserine	0	0
Taurine	0	0
Phospho ethanol amine	0	0
Urea	0	0
Aspartic acid	13.5	5.23
Threonine	7.07	3.70
Serine	7.48	3.04
Glutamic acid	38.6	12.93
Sarcosine	0	0
α-Amino asipic acid	0	0
Glycine	5.24	3.66
Alanine	27.59	12.23
Citrulline	1.10	1.10
α-Amino-n-butyric acid	0	0
Valine	14.76	7.16
Cystine	0	0
Methionine	3.94	1.20
Cystathionine	0.33	0.17
Isoleucine	10.90	4.22
Leucine	18.62	7.16
Tyrosine	3.14	1.83
Phenylalanine	11.93	4.84
β-Alanine	0.19	0.34
β-Amino isobutyric acid	0.87	0.75
γ-Amino-n-butyric acid	4.65	2.02
Ethanol amine	0	0.19
Ammonia	4.53	4.05
Hydroxylysine	0	0
Ornithine	2.97	1.00
Lysine	7.94	5.80
1-Methylhistidine	0	0
Histidine	1.25	0.95
3-Methylhistidine	0	0
Anserine	0	0
Carnosine	0	0
Arginine	8.83	3.78
Hydroxy proline	0	0
Proline	9.48	3.80

### Evaluation of the Growth Parameters of Rice Plants

Exposure to salt and drought stresses caused adverse effects on the morphological attributes of the plants. Characteristics such as root and shoot weight were significantly reduced by approximately 50–70% in the +ve control plants compared to the −ve control ones. The application of Si, KP, AR11, and biochar increased shoot weight by 15, 13, 31, and 12%, respectively, under salt stress, and co-application of Si + KP, Si + KP + AR11, Si + KP + Bio, and Si + KP + Bio + AR11 further enhanced this trait by 51, 77, 89, and 112% respectively. A similar increasing trend was observed for root weight. However, under drought stress, the sole or combined application of Si, KP, and biochar without AR11 produced only minor differences in the improvement of plant growth characteristics. Interestingly, these were significantly improved by the presence of AR11, both in the sole and combined application. The sole AR11 application enhanced shoot weight by 69%, and the co-application of Si + KP + Bio + AR11 increased it by 98%. Similarly, these same sole and combined applications increased root weight by 133 and 180%, respectively (Tables 4, 5). The results of study showed that the application of Si and KP along with biochar and *C. lunata* significantly improved the plants’ physiological and morphological attributes. Interesting results were observed under drought stress conditions, as it was shown that the growth-promoting effect of AR11 alone was more efficient compared to that produced by the combined application of biochar, Si, and K_2_HPO_4_.

**TABLE 4:** Effect of silicon, K_2_HPO_4,_ AR11, and biochar on morphological attributes under drought stress.

Treatments	Root Wt-D	Shoot Wt-D
Control1 −ve (Dr)	2.74 ± 0.23a	3.82 ± 0.2a
Control2 +ve (Dr)	0.71 ± 0.07ef	1.47 ± 0.01e
Si (Dr)	1.12 ± 0.13d	1.77 ± 0.04de
KP (Dr)	0.54 ± 0.31f	1.60 ± 0.08e
AR11 (Dr)	1.66 ± 0.26c	2.5 ± 0.15c
Bio (Dr)	0.82 ± 0.06ef	2.04 ± 0.55d
Si + KP (Dr)	0.80 ± 0.07ef	1.74 ± 0.07de
Si + KP + AR11 (Dr)	1.85 ± 0.08bc	2.73 ± 0.05bc
Si + KP + Bio (Dr)	0.90 ± 0.01de	1.80 ± 0.066de
Si + KP + Bio + AR11 (Dr)	1.99 ± 0.13b	2.92 ± 0.06b

*Each value represents the mean ± SD (n = 6). Different letters within the columns represent significant differences at P ≤ 0.05.*

**TABLE 5:** Effect of silicon, K_2_HPO_4,_ AR11, and biochar morphological attributes under salt stress.

Treatments	Root Wt.-S	Shoot Wt.-S
Control1 −ve (NaCl)	2.7 ± 0.11a	3.83 ± 0.25a
Control2 +ve (NaCl)	0.76 ± 0.12g	1.59 ± 0.11g
Si (NaCl)	0.96 ± 0.04f	1.84 ± 0.37f
KP (NaCl)	0.87 ± 0.04fg	1.81 ± 0.06f
AR11 (NaCl)	1.22 ± 0.06de	2.1 ± 0.1e
Bio (NaCl)	0.92 ± 0.04f	1.8 ± 0.1fg
Si + KP (NaCl)	1.16 ± 0.05e	2.42 ± 0.1d
Si + KP + AR11 (NaCl)	1.37 ± 0.05de	2.83 ± 0.06c
Si + KP + Bio (NaCl)	1.41 ± 0.11c	3.02 ± 0.15c
Si + KP + Bio + AR11 (NaCl)	2.04 ± 0.17b	3.4 ± 0.1b

*Each value represents the mean ± SD (n = 6). Different letters within the column represent significant differences at P ≤ 0.05.*

### Quantification of Mineral Elements in Plant Shoots

#### Phosphorous (P)

**FIGURE 2:**
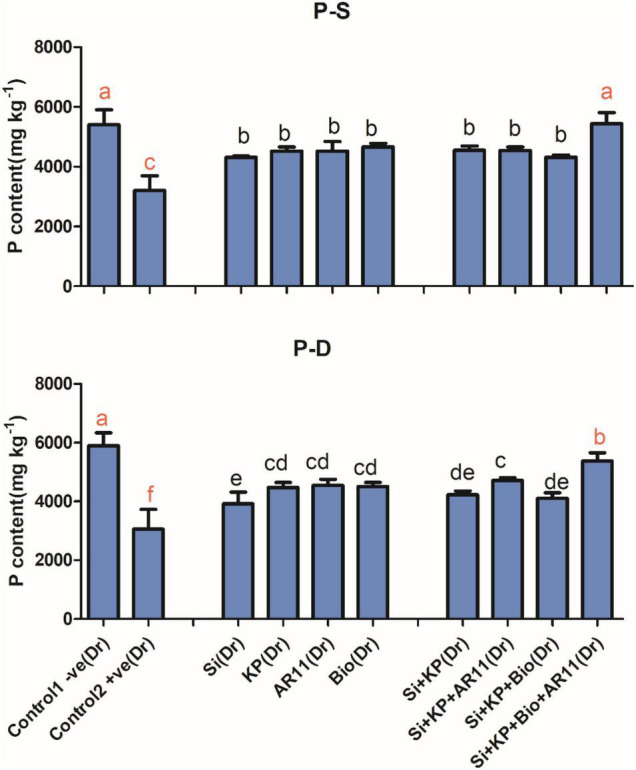
Effects of AR11, Si, K_2_HPO_4_, and biochar inoculation on phosphorus content in rice shoots subjected to salt and drought stresses. (-S), salt; (-D), drought. Error bars represent standard deviations. Each data point represents the mean of at least three replications. Bars with different letters are significantly different from each other at *P* ≤ 0.05.

Salt and drought stress conditions caused a significant drop in P content in the +ve control, specifically by 40 and 48%, respectively, when compared to the −ve control. Under drought stress, the sole application of Si, KP, AR11, and biochar improved P uptake by 28, 46, 49, and 47%, respectively. The co-application of Si + KP, Si + KP + AR11, Si + KP + Bio, and Si + KP + Bio + AR11 enhanced the P content of plant shoots by 38, 54, 34, and 76%, respectively. A similar trend was observed under salt stress, where the sole application of Si, KP, AR11, and biochar enhanced P uptake by 34, 40, 41, and 45%, respectively. The co-application of Si + KP, Si + KP + AR11, Si + KP + Bio, and Si + KP + Bio + AR11 increased P uptake by 41, 41, 34, and 69%, respectively, when compared to the +ve control (Figure 2).

#### Silicon (Si)

**FIGURE 3:**
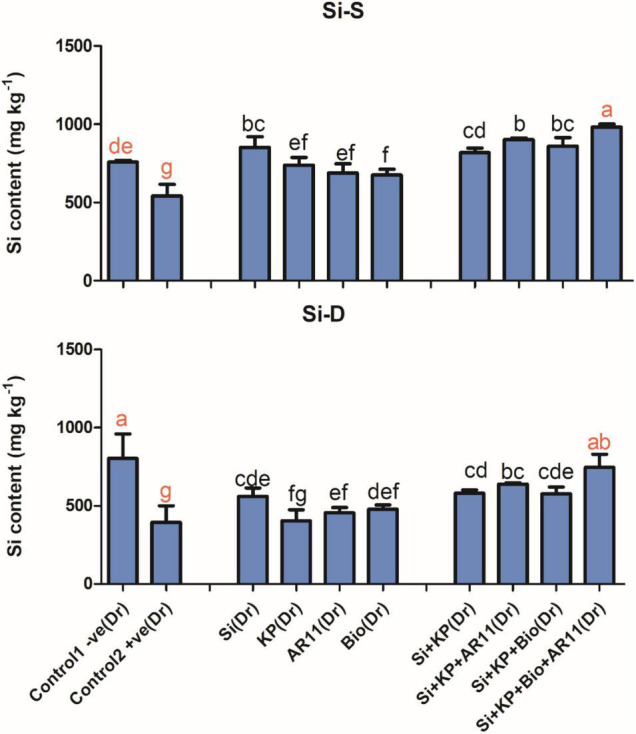
Concentration of silicon in rice shoots subjected to salt and drought stresses following AR11, Si, K_2_HPO_4_, and biochar inoculation. (-S), salt; (-D), drought. Error bars represent standard deviations. Each data point represents the mean of at least three replications. Bars with different letters are significantly different from each other at *P* ≤ 0.05.

The trend of Si uptake was similar to that of other elements. The Si content was significantly dropped under salt and drought stress in +ve control plants. However, the sole application of Si, KP, AR11, and biochar on increased Si content by 36, 27, 24, and 50%, respectively, under salt stress. Similarly, under drought stress, the sole application of Si, KP, AR11, and biochar enhanced Si content by 42, 2, 15, and 21%, respectively. The co-application of Si + KP, Si + KP + AR11, Si + KP + Bio, and Si + KP + Bio + AR11 increased Si content by 46%, 61%, 46% and 88%, respectively, under drought stress, and 50, 66, 58, and 80%, respectively, under salt stress when compared to the +ve control (Figure 3).

#### Sodium (Na)

**FIGURE 4:**
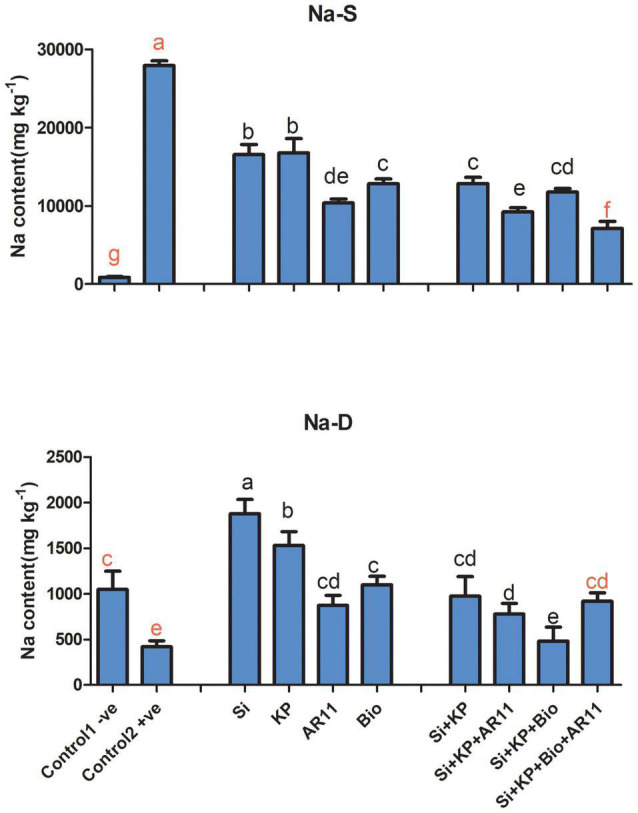
Influx of Na^+^ ion content in rice shoots subjected to salt and drought stresses following AR11, Si, K_2_HPO_4_, and biochar inoculation. (-S), salt; (-D), drought. Error bars represent standard deviations. Each data point represents the mean of at least three replications. Bars with different letters are significantly different from each other at *P* ≤ 0.05.

Under salt stress, Na^+^ ion content showed an approximate 27-fold increase in the +ve control compared to the −ve plants. However, treatment with Si, KP, AR11, and biochar lowered the ion content by 40, 39, 62, and 54%, respectively. Moreover, co-application caused a further decrease in Na^+^ ion content by 67, 57, and 74% in the plants treated with Si + KP + AR11, Si + KP + Bio, and Si + KP + Bio + AR11, respectively. Under drought stress, Na^+^ ion content dropped by 59% in the +ve control compared to the −ve control plants. Here, the plants treated with sole applications showed a higher Na^+^ ion uptake, compared to those treated with co-applications. The sole application of Si, KP, AR11, and biochar increased the Na^+^ ion level by 347, 264, 108, and 161%, respectively. However, co-application enhanced Na^+^ ion content less than the sole applications did—Si + KP (85%), Si + KP + AR11 (14%), Si + KP + Bio (118%), and Si + KP + Bio + AR11 (208%)—when compared to the +ve control plants (Figure 4). These results validate the detoxification effect of co-application of AR11, biochar, Si, and K_2_HPO_4_**.**

#### Potassium (K)

**FIGURE 5:**
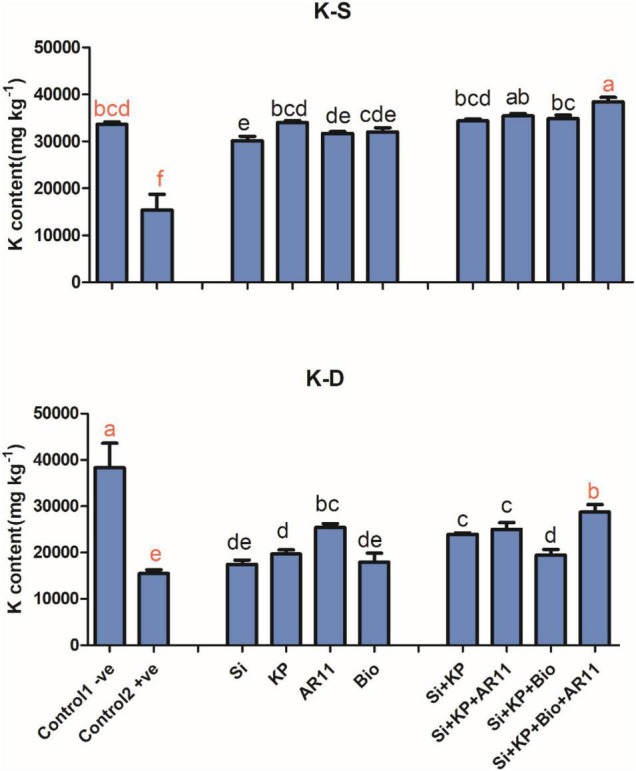
Quantification of K^+^ content in rice shoots subjected to salt and drought stresses following AR11, Si, K_2_HPO_4_, and biochar inoculation. (-S), salt; (-D), drought. Error bars represent standard deviations. Each data point represents the mean of at least three replications. Bars with different letters are significantly different from each other at *P* ≤ 0.05.

The sole application of Si, KP, AR11, and biochar significantly enhanced K content in both salt and drought stress conditions. Under salt stress, K content in the +ve control significantly dropped by 54%. However, the treatment with Si, KP, AR11, and biochar enhanced K uptake in plant shoots by 95, 120, 105, and 107%, respectively. The uptake of K was further stimulated by the co-application of Si + KP (122%), Si + KP + AR11 (130%), Si + KP + Bio (126%), and Si + KP + Bio + AR11 (149%). Under drought stress, K content significantly dropped by 59% in the +ve control compared to the −0ve control plants. However, the application of Si, KP, AR11, and biochar enhanced K uptake by 12, 27, 64, and 16%, respectively. The co-application of Si + KP, Si + KP + AR11, Si + KP + Bio, and Si + KP + Bio + AR11 further increased it by 54, 61, 25, and 85%, respectively, when compared to the +ve control (Figure 5). Indeed, the sole application of synthetic KP and biochar alone had a very small effect in mineral uptake under salt and drought stresses.

### Quantification of Abscisic Acid

Salt stress elevated the ABA level by 282% in +ve control plants compared to −0ve control. However, the ABA level significantly dropped by 59, 54, 60, 63, 65, 69, 61, and 69% with the treatment of Si, KP, AR11, Bio, Si + KP, Si + KP + AR11, Si + KP + Bio, and Si + KP + Bio + AR11 respectively.

**FIGURE 6:**
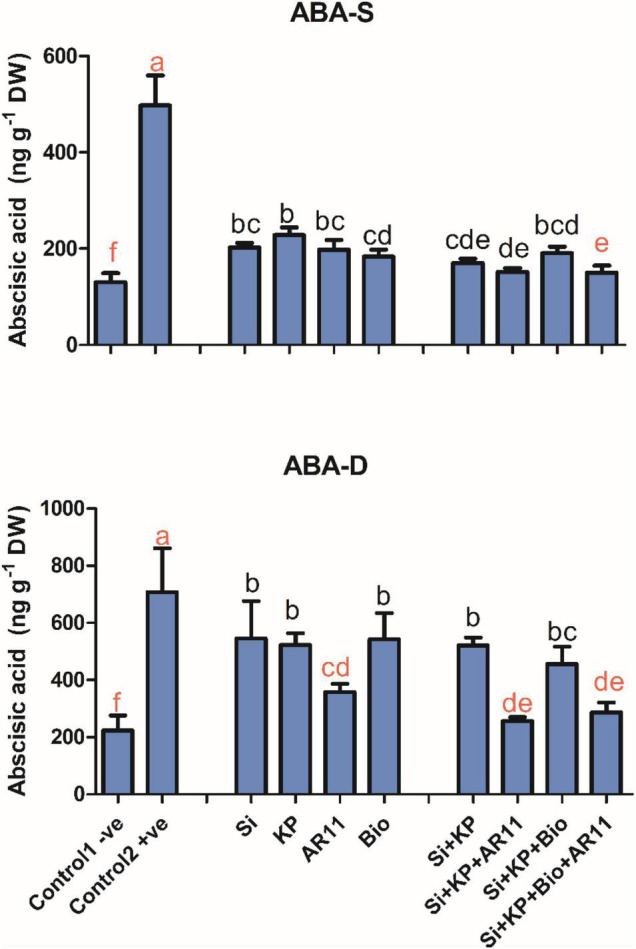
Effect of co-application (AR11, Si, K_2_HPO_4_, and biochar) on the abscisic acid (ABA) level under salt and drought stresses. (-S), salt; (-D), drought. Error bars represent standard deviations. Each data point represents the mean of at least three replications. Bars with different letters are significantly different from each other at *P* ≤ 0.05.

Similarly, under drought stress, the ABA level significantly increased by 216% in the +ve control compared to the −0ve control. Sole treatments with Si, KP, AR11, and biochar significantly dropped the ABA level by 22, 26, 49, and 23%, respectively, when compared to the +ve control plants. The co-application of Si + KP, Si + KP + AR11, Si + KP + Bio, and Si + KP + Bio + AR11 significantly reduced the ABA level by 26, 63, 35, and 59%, respectively (Figure 6).

### Analysis of Polyphenol Contents

**FIGURE 7:**
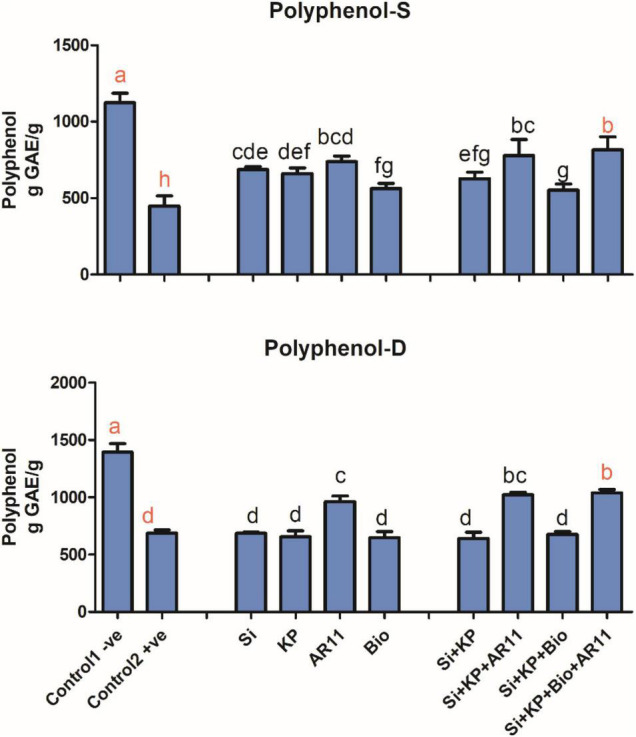
Effect of co-application (AR11, Si, K_2_HPO_4_, and biochar) on the phenolic level of plant shoots under salt and drought stresses. (-S), salt; (-D), drought. Error bars represent standard deviations. Each data point represents the mean of at least three replications. Bars with different letters are significantly different from each other at *P* ≤ 0.05.

Polyphenol contents in the +ve control plants significantly decreased by 50 and 60% under drought and salt stress, respectively. The application of Si, KP, AR11, and biochar under salt stress, significantly improved the polyphenol levels by 52, 46, 64, and 25%, respectively. However, minor differences were observed for the sole application of Si, KP, and biochar under drought stress. Interestingly, under drought stress, the sole application of AR11 produced higher polyphenol contents when compared with the co-application of Si + KP + Bio. Moreover, the co-application of Si + KP + Bio + AR11 significantly increased the polyphenol contents by 51%, compared to the +ve control (Figure 7).

### Proline Quantification

Proline content was significantly enhanced by 63 and 83% in +ve control plants exposed to salt and drought stresses, respectively, compared to the −0ve control. The sole application of Si, KP, AR11, and biochar decreased proline content by 14, 12, 24, and 15%, respectively, under salt stress when compared to +ve control. In addition, proline content dropped by 21, 31, 22, and 36% in plants shoot during treatment with Si + KP, Si + KP + AR11, Si + KP + Bio, and Si + KP + Bio + AR11, respectively, under salt stress.

**FIGURE 8:**
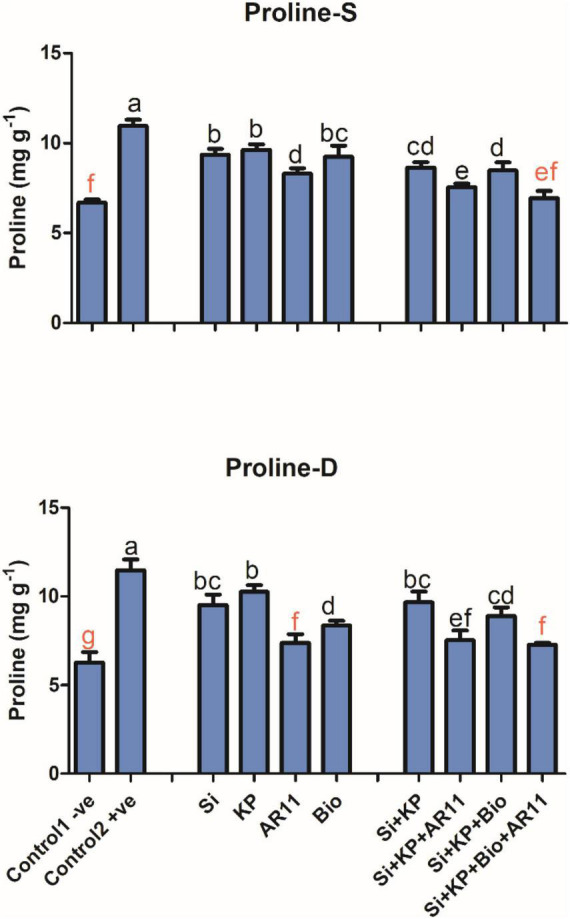
Analysis of proline content in plant shoots subjected to salt and drought stresses. (-S), salt; (-D), drought. Error bars represent standard deviations. Each data point represents the mean of at least three replications. Bars with different letters are significantly different from each other at *P* ≤ 0.05.

Similarly, the content decreased by 17, 10, 35, and 27% with the sole application of Si, KP, AR11, and biochar respectively, under drought stress. Moreover, proline content decreased by 15, 34, 22, and 36% in plants treated with Si + KP, Si + KP + AR11, Si + KP + Bio, and Si + KP + Bio + AR11, respectively under drought stress, compared to the +ve control (Figure 8).

### Analysis of Gene Expression in Rice Shoots

#### Potassium Transporter Gene *OsHKT2*

**FIGURE 9:**
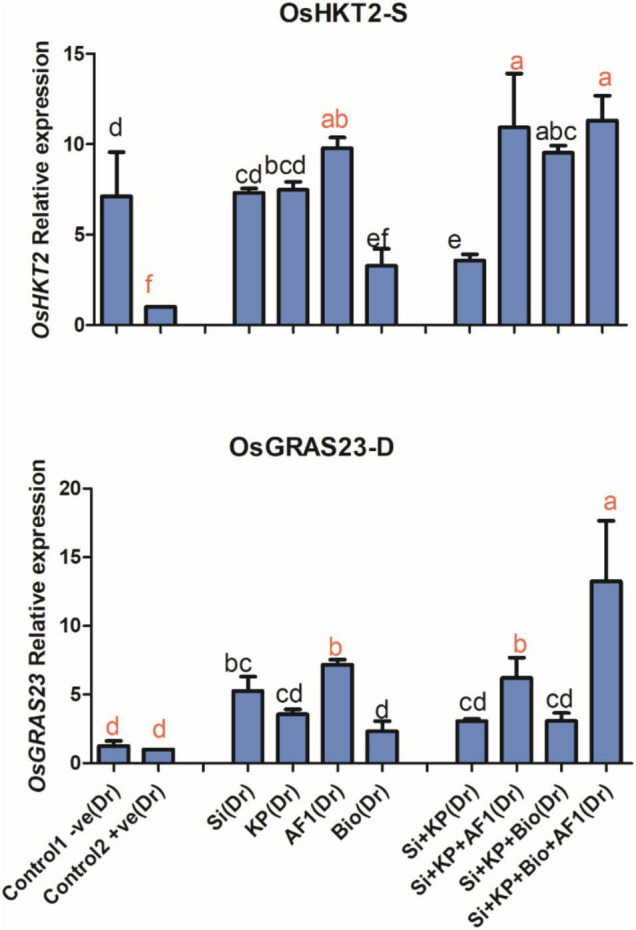
Expression level of the potassium transporter gene *OsHKT2* and drought-resistant *OsGRAS23* transcriptome in rice shoots subjected to salt and drought stresses. (-S), salt; (-D), drought. Error bars represent standard deviations. Each data point represents the mean of at least three replications. Bars with different letters are significantly different from each other at *P* ≤ 0.05.

The results of the present study showed that the K transporter gene *OsHKT2* was significantly downregulated under salt stress. However, the application of Si, KP, AR11, and biochar produced a 7, 7, 9, and 3-fold enhancement of its expression level, respectively, and co-application of Si + KP, Si + KP + AR11, Si + KP + Bio, and Si + KP + Bio + AR11, produced a further 3, 10, 9, and 11-fold expression enhancement respectively. These results validated the quantification of K content in rice shoots exposed to salt stress (Figure 9).

#### Drought-Resistant Gene *OsGRAS23*

The expression value of the drought-resistant *OsGRAS23* transcriptome in rice shoots treated with Si, KP, AR11, and biochar experienced a significant 5, 3, 7, and 2-fold enhancement, respectively. The co-application of Si + KP, Si + KP + AR11, Si + KP + Bio, and Si + KP + Bio + AR11, produced a further 3, 6, 3, and 13-fold increase in the expression level, respectively, when compared to the +ve control (Figure 9).

#### Silicon Transporter Gene *OsLsi1*

In the present study, the silicon transporter gene *OsLsi1* was highly expressed in the co-application of Si + KP + Bio + AR11. This is further confirmed by the high Si content observed in plants treated with co-application under both salt and drought stresses. Here, the application of Si, KP, AR11, and biochar caused a 10, 3, 8, and 5-fold upregulation of *OsLsi1* expression, respectively. The co-application of Si + KP, Si + KP + AR11, Si + KP + Bio, and Si + KP + Bio + AR11 stimulated a further 11, 13, 12, and 16-fold increase in the expression level of this gene, respectively, under salt stress.

**FIGURE 10:**
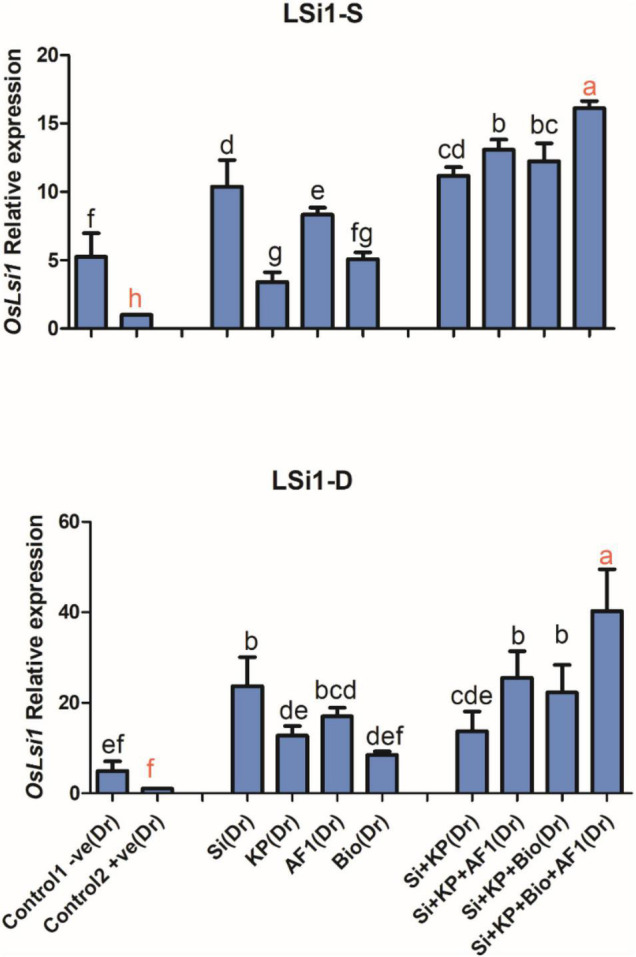
Expression level of the silicon-carrying gene *OsLSi1* in rice shoots subjected to salt and drought stresses following the sole and co-application of AR11, Si, K_2_HPO_4_, and biochar. (-S), salt; (-D), drought. Error bars represent standard deviations. Each data point represents the mean of at least three replications. Bars with different letters are significantly different from each other at *P* ≤ 0.05.

A similar trend was observed under drought stress, where *OsLsi1* expression experienced 3, 12, 17, and 8-fold increase during treatments with Si, KP, AR11, and biochar, respectively. The plants treated with Si + KP, Si + KP + AR11, Si + KP + Bio, and Si + KP + Bio + AR11 co-applications showed a 13, 25, 22, and 40-fold increase in *OsLsi1* expression, respectively (Figure 10).

## Discussion

There is an urgent need to counteract the effects of environmental stresses, chemical pollution, climate change, and economic inflation on agriculture (Arif et al., 2019; Gupta et al., 2020). As biostimulants (biochar and PGPMs) are widely reported as ecologically safe agents for enhancing the efficiency of inorganic fertilizers and mitigating environmental stresses (Grobelak et al., 2015; Hossain et al., 2017; Zou et al., 2021), in the present study we elucidated how these agents mobilize synthetic Si, P, and K contents and modulate the internal physiology of rice plants exposed to drought and salt stress conditions. The use of PGPMs and biochar is a promising tool for crop improvement; biochar creates favorable condition for sustaining microbiomes which facilitates symbiotic association of PGPMs in plant rhizosphere (Zhang et al., 2018; Gorovtsov et al., 2020). As PGPMs are widely reported for producing secondary metabolites and mediating nutrient recycling; they would further intensify the nutrient assimilation that maintains various metabolic processes and confer stress tolerance in crops (Egamberdieva et al., 2018; Khan et al., 2019b; Kazerooni et al., 2021). In our experimental process, we observed combined application of Si, P, K, AR11, and biochar produced a symbiotic effect that downregulated the Na influx and enhanced the uptake of K, Si, and P contents by plants, therefore maintaining the ionic balance in their metabolic system.

Excessive salt concentrations cause ionic disturbances in plants, and drought further exacerbates the effect of salinity on crops causing osmotic stress, which may ultimately lead to cell death (Sahin et al., 2018). The results of the current study showed that the plant growth was tremendously improved with the combined application of Si, PK, AR11, and biochar. Previous study has reported that GA producing microbes could play a key role in regulating endogenous phytohormones under salt and drought stress (Khan et al., 2015). Additionally, organic acid chelates ions, dissolutes the insoluble mineral ions and enhance bioavailability to plant uptake (Ullah et al., 2015). Since the *C. lunata* AR11 strain has the potential to spontaneously produce GA (GA_1_, GA_3_, GA_4_, and GA_7_), and organic acids that can possibly boost the uptake of essential elements under salt and drought stress conditions.

The researchers are now desperately conscientious in developing the approach of optimizing fertilizer use efficiency and mitigating stress for sustainable agriculture. Despite the efforts, limited success has been achieved in terms of improving the utilization efficiency of synthetic fertilizers (Tounkara et al., 2020). Thus, the main goal of the present study was based on enhancement of nutrient use efficiency. In our study, the application of AR11 and biochar significantly increased the uptake of Si, P, and K in rice plants. Ma et al. (2006) first succeeded in revealing a suppression of *OsLsi1* results in reduced silicon uptake in rice. Moreover, Ma et al. (2007) further discovered efflux transporter of silicon *OsLsi2* which revealed a unique phenomenon in nutrient transport in rice. Thus, a higher uptake values of Si in our results were further correlated with the higher expression of the Si transporter gene *OsLsi1*.

Moreover, the ABA stress hormone was observed to be low in the plants treated with co-application, under both stress conditions. As ABA regulates stomatal conductance, which in turn regulates the transpiration rate in plants, a higher ABA level or depletion represents a higher stress level (Chen et al., 2020; González-Guzmán et al., 2021). The results obtained in this study indicate the low-stress level present in plants, which is further validated by the higher expression of the stress-responsive genes *OsGRAS23* and *OSHKT2*. Our results are supported by various previous studies which have shown that the application of exogenous Si, K_2_HPO_4_, biochar, and plant growth-promoting microorganisms improved Si, K, and P contents and *OsLsi1* expression and decreased the ABA level in plants under abiotic stress (Kim et al., 2014; Adhikari et al., 2020b; Khan et al., 2020). These results are further supported by findings reported in various previous studies, for example, Gong et al. (2006) and Gengmao et al. (2015) showed that Si reduced Na^+^ uptake under salt stress. The combined effect of biochar and silicon was shown to improve several physiological traits, like seed yield and oil quality, in *Helianthus annuus* under water deficit stress (Seleiman et al., 2019).

Reactive oxygen species (ROS), a by-product of aerobic metabolism are recognized as oxygen radicals which are highly vulnerable for normal plant metabolic processes (Zaid and Wani, 2019). To neutralize these negative effects, plants activate antioxidant system to produce phenolic content (Bilal et al., 2018; Decros et al., 2019). Our results showed that AR11 application combined with biochar, silicon, and potassium phosphate significantly increased polyphenol contents. These results agree with Li et al. (2012), Hashem et al. (2015), and Ikram et al. (2018) who reported the role of biostimulants in strengthening the antioxidant system of plants under abiotic stresses.

Altogether, our results coincide with the findings of several authors: Danish and Zafar-ul-Hye (2019) showed biochar and PGPR co-application improved yield of wheat under drought stress, Asaf et al. (2018) reported GA producing fungus *Aspergillus flavus* conferred salt tolerance in *Glycine max*. Similarly, Biju et al. (2017) and Cui et al. (2021) reported Si application alleviated drought stress in crops by regulating enzymes, osmolytes, oxidative metabolism, and nutrient assimilation. Adnan et al. (2020) and Kaya et al. (2003) showed the supplementation of potassium and phosphorus coupled with phosphate solubilizing bacteria ameliorated salt stress in maize, pepper and cucumber plants. These evidence suggest that our methodology covers the magnitude of all of these stress mitigation tools with a single comprehensive approach.

## Conclusion

To sum up, our study demonstrated that the co-application of Si (2 mM), K_2_HPO_4_ **(**3 mM) and biochar (3%) with isolate AR11 could significantly optimize the nutrient uptake efficiency of plants, modulate plant growth regulators and stress-responsive genes to confer salt (150 mM) and drought (20–40%) of soil moisture level] stress tolerance in rice. The strategies defined in this study could contribute to reducing the use of fertilizers, minimizing production costs and environmental pollution, decreasing food toxicity, and promoting sustainable agriculture.

## Data Availability Statement

The datasets presented in this study can be found in online repositories. The names of the repository/repositories and accession number(s) can be found at: https://www.ncbi.nlm.nih.gov/ and https://www.ncbi.nlm.nih.gov/nuccore/MZ145250.1/.

## Author Contributions

AA conceptualized the research and methodologies, conducted the experiment, and prepared the original draft. MAK, MI, S-MK, and K-EL helped in investigation. G-JJ and JYS provided the resource for investigation and managed the software. B-WY reviewed and edited the manuscript. S-MK and I-JL supervised, administered, and managed the funding. All authors contributed to the article and approved the submitted version.

## Conflict of Interest

The authors declare that the research was conducted in the absence of any commercial or financial relationships that could be construed as a potential conflict of interest.

## Publisher’s Note

All claims expressed in this article are solely those of the authors and do not necessarily represent those of their affiliated organizations, or those of the publisher, the editors and the reviewers. Any product that may be evaluated in this article, or claim that may be made by its manufacturer, is not guaranteed or endorsed by the publisher.
