# Supplementary material for: The Combined Inoculation of Curvularia lunata AR11 and Biochar Stimulates Synthetic Silicon and Potassium Phosphate Use Efficiency, and Mitigates Salt and Drought Stresses in Rice
Source: Front Plant Sci. 2022 Mar 3;13:816858. doi: 10.3389/fpls.2022.816858 (PMC8928408; doi:10.3389/fpls.2022.816858)

Table 1. Screening of plant growth promoting traits of fungal isolates. which showed the higher potential of functional role under stress.

| **Fungal isolates** | **Cd-R** | **Cr-R** | **Ni-R** | **Cu-R** | **Zn-R** | **MNF-Col** | **SOP-Col** | **Bio-Col** | **F+Bio-symbiosis** | **GA-P** | **OA-P** | **NaCl-R** |
| --- | --- | --- | --- | --- | --- | --- | --- | --- | --- | --- | --- | --- |
| **AR11** | ******* | ******* | ****** | ******* | ******* | ****** | ******* | ******* | ******* | ******* | ******* | ******* |
| **AF7** | ****** | ****** | ****** | ******* | ****** | ******* | ******* | ******* | ******* | ******* | ******* | ******* |
| AR13 | * | ** | * | ** | * | * | ** | * | * | ND | * | * |
| AR17 | * | ** | * | * | ** | * | * | * | * | ND | * | * |
| AR22 | * | * | * | * | * | * | * | ** | * | ND | ** | * |
| AR29 | * | * | * | * | * | * | * | * | * | ND | * | * |
| AR48 | ND | * | * | * | * | * | * | * | * | ND | ** | * |
| AR56 | ND | * | * | ** | ** | * | * | * | ** | ND | * | * |
| AR59 | ND | * | * | ** | ** | * | * | * | ** | * | * | * |
| AR113 | ND | * | * | * | ** | ND | * | * | * | * | ** | * |
| AF26 | ND | ND | ND | ** | ** | ** | * | * | * | * | * | * |
| AF32 | ND | ND | ND | * | ** | * | * | * | * | * | * | * |
| AF43 | ND | ND | * | ** | ** | ND | * | * | * | * | * | * |

***(-HIGH), **(-Moderate), *(- Low), ND: Not Detected, -R: Resistant MNF-Col: Mineral nutrient fertilizer colonization, SOP-Col: Sulphate of Potash Colonization, Bio-Col: Biochar Colonization, F+Bio: Fertilizer+Biochar Colonization, GA-P: Gibberellin Production, OA-P: Organic acid production, NaCl: Salt resistant


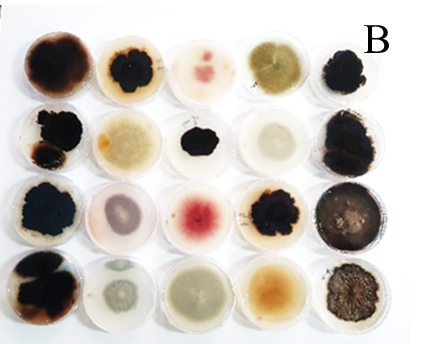


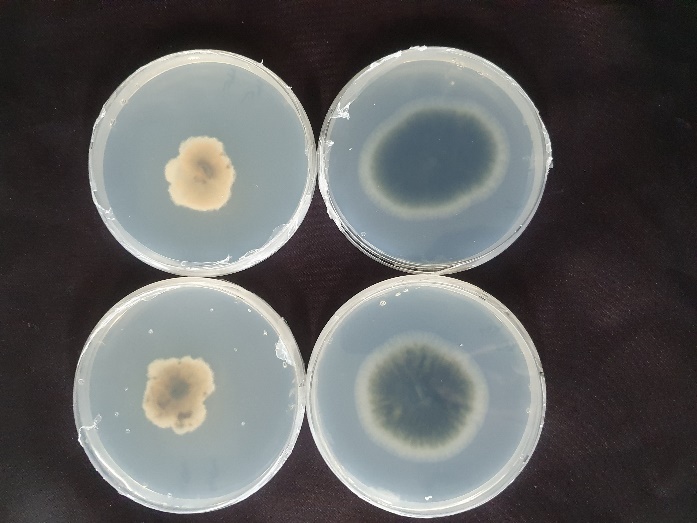


**B**

**A**

**Figure 1. Isolation and selection of plant growth promoting rhizoshperic fungal isolates(A), Salt resistivity test of AR11 isolate (B)**


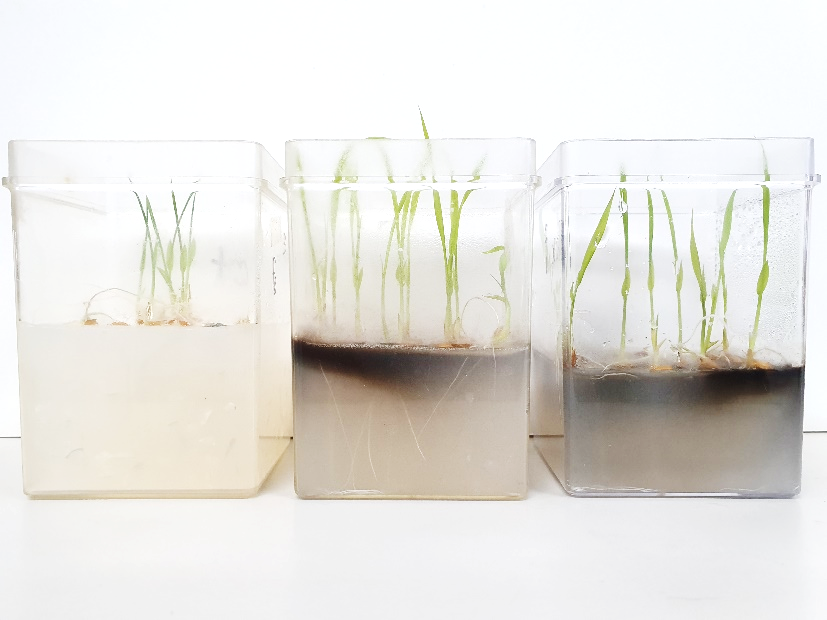


Control AR11 AF7

Figure 2. Detecting gibberellin producing ability of isolate AR11 and AF7 through observing the growth of rice mutant *Waito-C* prior to gibberellin quantification in pure culture

Table 2. List of primers used for gene expression analysis

| Genes | Forward Primer | Reverse Primer |
| --- | --- | --- |
| GmST1 | 5’TCTAGAATGGCGTTTGTTGCAGCCATG3’ | 5’GAGCTCTCATAAGGTTCGGGGATCCTTTC3’ |
| GmAKT2 | 5’CACCATGCTAGCTGATCGTTACC3’ | 5’TCAGCTTATCCAACAAAAAAAAAGT3’ |
| GmSALT3 | 5’ATGACGTTCAACGCGAGC3’ | 5’AAGTTCTTCGATAGCATCTTTA3’ |


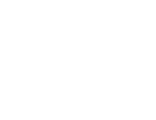

Supplement: Supplementary file 1 [file Data_Sheet_1.docx]
